# ITGAV as a promising diagnostic, immunological, and prognostic biomarker in pan-cancer

**DOI:** 10.1038/s41598-025-11836-8

**Published:** 2025-08-07

**Authors:** Hanyang Su, Jie Wang, Xinyu Cao, Xiangqian Zhang, Huajun Zhang, Xiaojin Liu

**Affiliations:** 1 Department of Respiratory Medicine & Teaching and Research Section of Clinical Nursing, Xiangya Hospital, Central South University, Changsha, China; 2 Department of Physiology, Xiangya School of Basic Medical Sciences, Central South University, Changsha, China; 3 Department of Oncology, National Clinical Research Center for Geriatric Disorders, Xiangya Hospital, Central South University, Changsha, 410008 Hunan China; 4 Department of Ultrasonic Imaging, Xiangya Hospital, Central South University, Changsha, 410008 Hunan China; 5 Department of Oncology, NHC Key Laboratory of Cancer Proteomics & State Local Joint Engineering Laboratory for Anticancer Drugs, National Clinical Research Center for Geriatric Disorders, Xiangya Hospital, Central South University, Changsha, 410008 Hunan China; 6 Department of Plastic and Cosmetic Surgery, Xiangya Hospital, Central South University, Changsha, 410008 Hunan China

**Keywords:** ITGAV, Pan-cancer, Prognosis, Immunotherapy, Immune infiltration, Biomarker, Cancer, Computational biology and bioinformatics, Oncology

## Abstract

Integrin αV (ITGAV) plays a key role in cell adhesion, migration, and immune regulation, and is implicated in tumor progression. However, its comprehensive expression profile and functional relevance across different cancers remain poorly understood. We conducted an integrative pan-cancer analysis of ITGAV using data from TCGA, GTEx, CCLE, and other public databases. Expression, diagnostic value (via ROC analysis), and prognostic significance (via Cox and Kaplan–Meier analyses of OS, DSS, PFS, and DFS) were assessed. We further explored ITGAV’s correlation with immune cell infiltration and immune-related genes, its predictive role in immunotherapy response based on immunophenoscore (IPS), and its drug-binding potential through molecular docking. (1) ITGAV was significantly overexpressed in multiple cancer types including LIHC, COAD, and STAD. (2) ROC analysis confirmed its strong diagnostic value, particularly in HNSC, UCEC, and ESCA. (3) High ITGAV expression was associated with poorer survival outcomes in most cancers, while a protective role was observed in KIRC. (4) ITGAV expression was positively correlated with immune cell infiltration and co-expressed with immune-activating and immunosuppressive genes. (5) The expression level of ITGAV correlates with the IPS score, suggesting its predictive value for the benefit of immunotherapy. (6) Molecular docking identified strong binding affinities between ITGAV and six candidate compounds, including gemcitabine and pioglitazone. Our findings demonstrate that ITGAV is a promising biomarker for diagnosis, prognosis, and immunotherapy prediction across cancers. Its immunological associations and druggability highlight its potential as a candidate therapeutic target.

## Introduction

Immunotherapy, particularly immune checkpoint blockade with agents such as PD-1, PD-L1, and LAG-3 antagonists, has revolutionized cancer treatment and has shown remarkable clinical benefits in certain patients^1^. However, the majority of cancer patients do not respond to these therapies, and the development of resistance is common^2,3^. The tumor microenvironment (TME) and extracellular matrix (ECM) are key determinants of tumor growth and can significantly impact the response to immunotherapy by modulating the behavior of immune cells^4,5^. Despite the promise of targeting the TME and ECM in cancer treatment, the complex mechanisms regulating their formation and dynamics pose a challenge^6,7^. Thus, to improve current immunotherapies, comprehensively investigating the genes involved in the TME and ECM is essential.

Integrins are a group of heterodimeric transmembrane proteins composed of 18 alpha subunits and 8 beta subunits that play crucial roles in cell adhesion and communication between cells^8,9^. ITGAV, also known as integrin alpha subunit V (CD51), is a member of the integrin alpha chain family. ITGAV binds to β1, β3, β5, β6, and β8 to form a heterodimer and is involved in the regulation of fibronectin, bilirubin, and fibrinogen receptors^10^. Linearly linked integrins can activate multiple kinase pathways and their respective effectors^11–13^. These pathways include numerous signaling pathways, such as the FAK/Src, PI3K/Akt, and MAPK/ERK pathways, which are essential for tumor cell proliferation, migration, and invasion^14–16^. Hence, increasing evidence suggests that overexpression of ITGAV is associated with tumor progression and poor prognosis in various malignancies, including glioblastoma^17^, hepatocellular carcinoma^18^, breast cancer^19^, colorectal cancer^20^, and lung cancer^21^; gastric cancer^22^; prostate cancer^23^; and head and neck squamous cell carcinoma^24^. ITGAV expression is correlated with tumor immune cells, indicating its potential as an immunotherapeutic target^25,26^. However, ITGAV-targeted therapies have yielded limited clinical efficacy^27,28^, and the precise immune-modulatory mechanisms remain unclear. While ITGAV has been studied in specific cancers, existing literature primarily focuses on individual tumor types and lacks integrative immune-genomic and therapeutic-level evaluations. Importantly, prior failures in clinical trials may stem from the absence of mechanistic context and patient stratification, rather than from the inadequacy of ITGAV as a therapeutic target.

To address these limitations, we conducted the first comprehensive pan-cancer analysis of ITGAV by integrating transcriptomic, immunological, and pharmacological data from 33 tumor types. This study systematically investigates ITGAV’s differential expression, prognostic and diagnostic potential, associations with immune cell infiltration and immune-related gene networks, predictive value for immunotherapy efficacy, and druggability based on molecular docking simulations. Unlike prior studies limited to expression profiling in isolated cancer contexts, our multi-dimensional approach aims to provide an integrative understanding of ITGAV’s biological and immunological roles across cancer types. These findings offer valuable insights into its potential as a biomarker and therapeutic target for personalized immuno-oncology. This multi-dimensional approach provides a more comprehensive view of ITGAV’s functional roles in cancer, particularly in relation to the tumor immune microenvironment, and supports its reevaluation as a biomarker and potential therapeutic target in precision oncology.

## Methods

### Analysis of ITGAV expression and localization

To comprehensively analyze the expression profile of ITGAV across human cancers, we integrated data from several publicly available databases. First, we used TIMER (Tumor Immune Estimation Resource) to examine ITGAV expression in tumor versus adjacent normal tissues across multiple cancer types. TIMER applies a standardized analytical pipeline, including batch correction and immune deconvolution, to ensure uniform cross-tissue comparisons based on TCGA data^29^. To complement these results, we also downloaded raw HTSeq-FPKM expression data from the TCGA Genomic Data Commons (GDC). Independent preprocessing—including normalization and log₂ transformation—was performed using R software (version 4.2.1) following TCGA RNA-seq analysis standards^30^. Additionally, we used the Broad Institute Cancer Cell Line Encyclopedia (CCLE) to analyze ITGAV expression in multiple cancer cell lines^31^. For tissue-specific expression analysis, we utilized the Genotype-Tissue Expression (GTEx) project dataset^32^. To explore the localization of the ITGAV protein, the Human Protein Atlas (THPA) database was used to provide information on RNA expression and subcellular localization across different cell lines^33^.

### Correlation analysis of ITGAV expression with diagnosis, clinical stage and prognosis in pan-cancer

In this study, we systematically evaluated the diagnostic and prognostic significance of ITGAV expression across various cancer types. To assess its diagnostic potential, receiver operating characteristic (ROC) curve analyses were performed using TCGA datasets for multiple tumor types. The area under the curve (AUC) was calculated to quantify diagnostic performance. To investigate the association between ITGAV expression and tumor clinical stage, we applied the Kruskal–Wallis test to compare expression levels across different pathologic stage. All statistical analyses and data visualizations were conducted using R software (version 4.2.1), with plotting performed via the ggplot2 package (version 3.5.1)^34^. For prognostic evaluation, we explored the association of ITGAV expression with overall survival (OS), progression-free survival (PFS), disease-free survival (DFS), and disease-specific survival (DSS) using univariate Cox proportional hazards regression models. The statistical significance of survival differences was assessed using log-rank tests, and a *p-value* < 0.05 was considered statistically significant.

### Functional enrichment and interaction network analysis of ITGAV

To explore the protein–protein interaction (PPI) network of ITGAV, we employed the GeneMANIA database, which predicts functionally associated genes based on various data sources, including co-expression, co-localization, and physical interactions^35^. This analysis allowed us to identify potential interacting partners of ITGAV and provided insights into its putative biological functions and roles in carcinogenesis. Based on the ITGAV-centered gene interaction network, we further performed Gene Ontology (GO) enrichment analysis and Kyoto Encyclopedia of Genes and Genomes (KEGG) pathway analysis to investigate the biological processes, cellular components, molecular functions, and signaling pathways associated with ITGAV and its interacting genes.

### Correlation analysis of ITGAV expression with immune infiltration across cancers

To investigate the association between ITGAV expression and immune cell infiltration in pan-cancer, we utilized the TIMER database to assess correlations between ITGAV expression and the estimated infiltration levels of six major immune cell types: CD8⁺ T cells, CD4⁺ T cells, macrophages, dendritic cells, neutrophils, and B cells^36^. Six representative cancer types were selected for detailed visualization.

In addition, we explored the relationship between ITGAV expression and a broad panel of immune-related genes, including genes encoding immune activators, immune suppressors, immune checkpoint molecules, major histocompatibility complex (MHC) proteins, chemokines, and chemokine receptors. These analyses provide insight into the immunological landscape associated with ITGAV expression across different tumor types. Data visualization was performed using the R packages reshape2 (version 1.4.4) and RColorBrewer (version 1.1.3)^37^ to generate correlation heatmaps.

### Molecular docking of drugs with ITGAV

Drug–gene interaction data were obtained from the DSigDB database^38^. Subsequently, drug enrichment analysis was performed using the R packages enrichplot (version 1.24.4) and ggplot2 (version 3.5.1)^34^for visualization. The 2D and 3D chemical structures of candidate small-molecule drugs were retrieved from the PubChem database^38^, while the three-dimensional structure of the ITGAV protein was obtained from the Protein Data Bank (PDB)^39^. For molecular docking analysis, the structural data of both the target protein and small-molecule compounds were uploaded to the CB-DOCK2 platform. Among the predicted docking conformations, the binding mode with the lowest binding free energy was selected and visualized to characterize potential binding interactions between ITGAV and the candidate drugs^40^.

### Correlation analysis of ITGAV expression with sensitivity to immunotherapy across cancers

To evaluate the potential association between ITGAV expression and immunotherapy response across cancer types, immunophenoscores (IPS) were obtained from The Cancer Immunome Atlas (TCIA) database. The IPS represents a computationally derived metric that reflects tumor immunogenicity and predicts potential responsiveness to immune checkpoint inhibitors (ICIs). IPS data were extracted for multiple TCGA cancer types and stratified by high and low ITGAV expression groups based on median expression values. The analysis focused on four ICI treatment scenarios: CTLA-4⁺/PD-1⁺, CTLA-4⁻/PD-1⁺, CTLA-4⁺/PD-1⁻, and CTLA-4⁻/PD-1⁻. All statistical tests were two-sided, and a p-value < 0.05 was considered statistically significant.

### Statistical methods

Statistical analyses were performed via R software (version 4.2.1). Spearman’s correlation and the Kruskal‒Wallis test were used to assess the relationships between ITGAV expression and clinicopathological features, as well as immune infiltration across different cancer types. Survival analysis, including OS, PFS, DFS, and DSS, was conducted via Cox regression models and Kaplan‒Meier curves, with the significance level set at p < 0.05. Diagnostic accuracy was evaluated via receiver operating characteristic (ROC) curves and the area under the curve (AUC). Co-expression analysis of ITGAV with immune-related genes was performed. The IPS was analyzed to assess immunotherapy sensitivity, with statistical significance determined by the Kruskal‒Wallis test. All tests were two-sided, with p < 0.05 considered statistically significant.

## Results

### Altered expression of ITGAV across cancers and cell lines

**Fig. 1:**
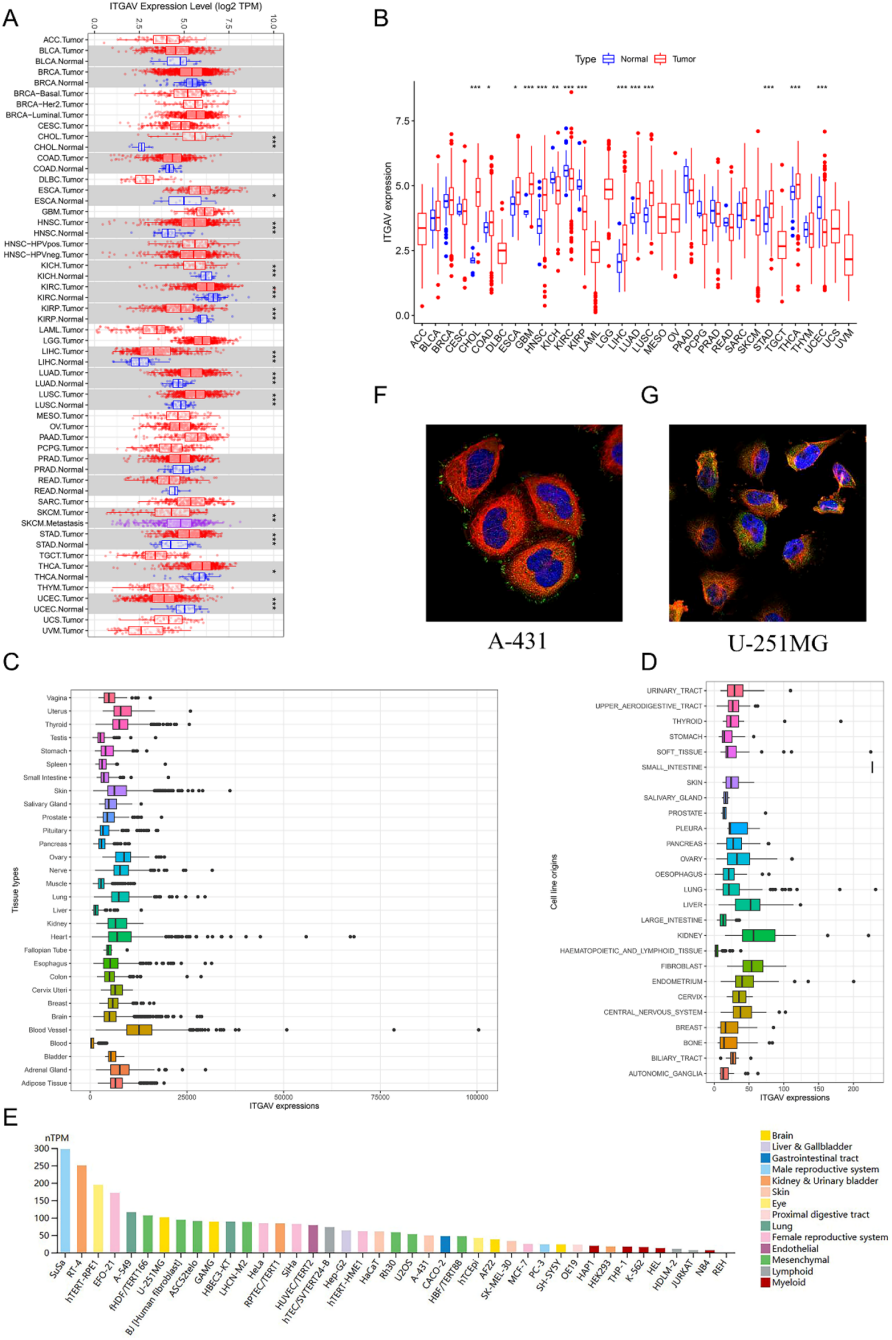
ITGAV expression and localization in pan-cancer. (**A**) Pan-cancer analysis of ITGAV expression showing elevated levels in CHOL, ESCA, and HNSC. (**B**) Validation of ITGAV overexpression using TCGA data, especially in CESC and GBM. (**C**) Tissue-specific expression of ITGAV based on GTEx data, with higher expression in skin, and vascular tissues, and lower levels in spleen, pancreas, liver, and blood. (**D**) Expression levels of ITGAV across cancer cell lines from the CCLE dataset, with prominent expression in ovarian, lung, liver, and kidney cell lines. (**E**) RNA expression levels of ITGAV in different cell lines, with highest expression observed in SuSa, RT-4, and hTRET-RPE1 cells. (**F-G**) Immunofluorescence staining in A-431 and U-251MG cells, demonstrating ITGAV protein localization in the cytoplasm and membrane. Nuclei were counterstained with DAPI (blue), and ITGAV was visualized in green.

ITGAV expression was analyzed across multiple cancer types via data from the TIMER, TCGA, GTEx, and CCLE databases. The TIMER data revealed that ITGAV expression was significantly elevated in various cancer tissues, including cholangiocarcinoma (CHOL), esophageal carcinoma (ESCA), head and neck squamous cell carcinoma (HNSC), liver hepatocellular carcinoma (LIHC), lung adenocarcinoma (LUAD), lung squamous cell carcinoma (LUSC), stomach adenocarcinoma (STAD) and thyroid carcinoma (THCA) (Fig. 1A). This finding was further validated by TCGA data, which revealed significant overexpression of ITGAV in CHOL, colorectal cancer (COAD), ESCA, glioblastoma multiforme (GBM), HNSC, LIHC, LUAD, LUSC, STAD, and THCA (Fig. 1B). Tissue-specific expression analysis of the GTEx database revealed that ITGAV expression was relatively high in skin, and vascular tissues, whereas its expression was relatively low in the spleen, pancreas, liver, and blood (Fig. 1C). Similarly, analysis of CCLE data indicated that tumor cell lines derived from tissues such as ovaries, livers, kidneys, and fibroblasts expressed relatively high levels of ITGAV, whereas those derived from the small intestine and prostate expressed relatively low levels (Fig. 1D). Additionally, RNA expression patterns revealed that ITGAV was notably expressed in the SuSa, RT-4, and hTRET-RPE1 cell lines (Fig. 1E). Immunofluorescence staining of A-431 and U251MG cells revealed that the ITGAV protein was predominantly localized to the cytoplasm and cell membrane (Fig. 1F-G).

### ITGAV Shows Strong Diagnostic Potential in Pan-Cancer

**Fig. 2:**
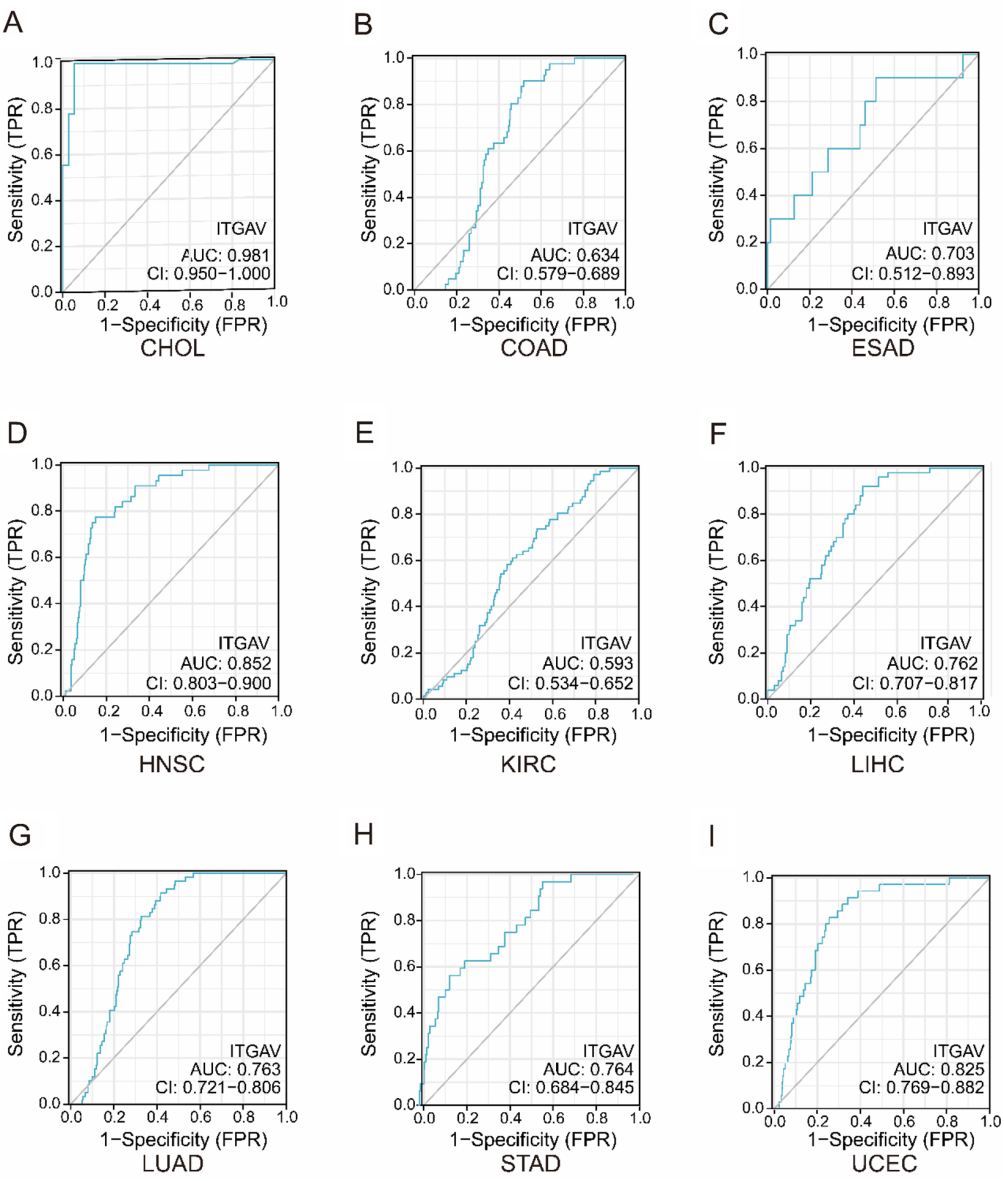
Diagnostic performance of ITGAV across cancers. (**A**–**I**) ROC curves assessing the diagnostic value of ITGAV in various tumor types, including CHOL, COAD, ESAD, HNSC, KIRC, LIHC, LUAD, STAD, and UCEC. AUC values are provided for each cancer type, reflecting diagnostic accuracy based on TCGA expression data.

As illustrated in Fig. 2A–I, ITGAV showed strong diagnostic performance in CHOL (AUC = 0.981, Fig. 2A), HNSC (AUC = 0.852, Fig. 2D), and UCEC (AUC = 0.825, Fig. 2I). Moderate diagnostic ability was observed in LIHC (AUC = 0.762, Fig. 2F), LUAD (AUC = 0.763, Fig. 2G), and STAD (AUC = 0.764, Fig. 2H). In contrast, ITGAV demonstrated relatively lower diagnostic performance in COAD (AUC = 0.634, Fig. 2B), ESAD (AUC = 0.703, Fig. 2C), and KIRC (AUC = 0.593, Fig. 2E). These results suggest that ITGAV holds potential as a diagnostic biomarker in multiple cancers, particularly in CHOL, HNSC, and UCEC.

### ITGAV expression is significantly associated with clinicopathological features across cancers

**Fig. 3:**
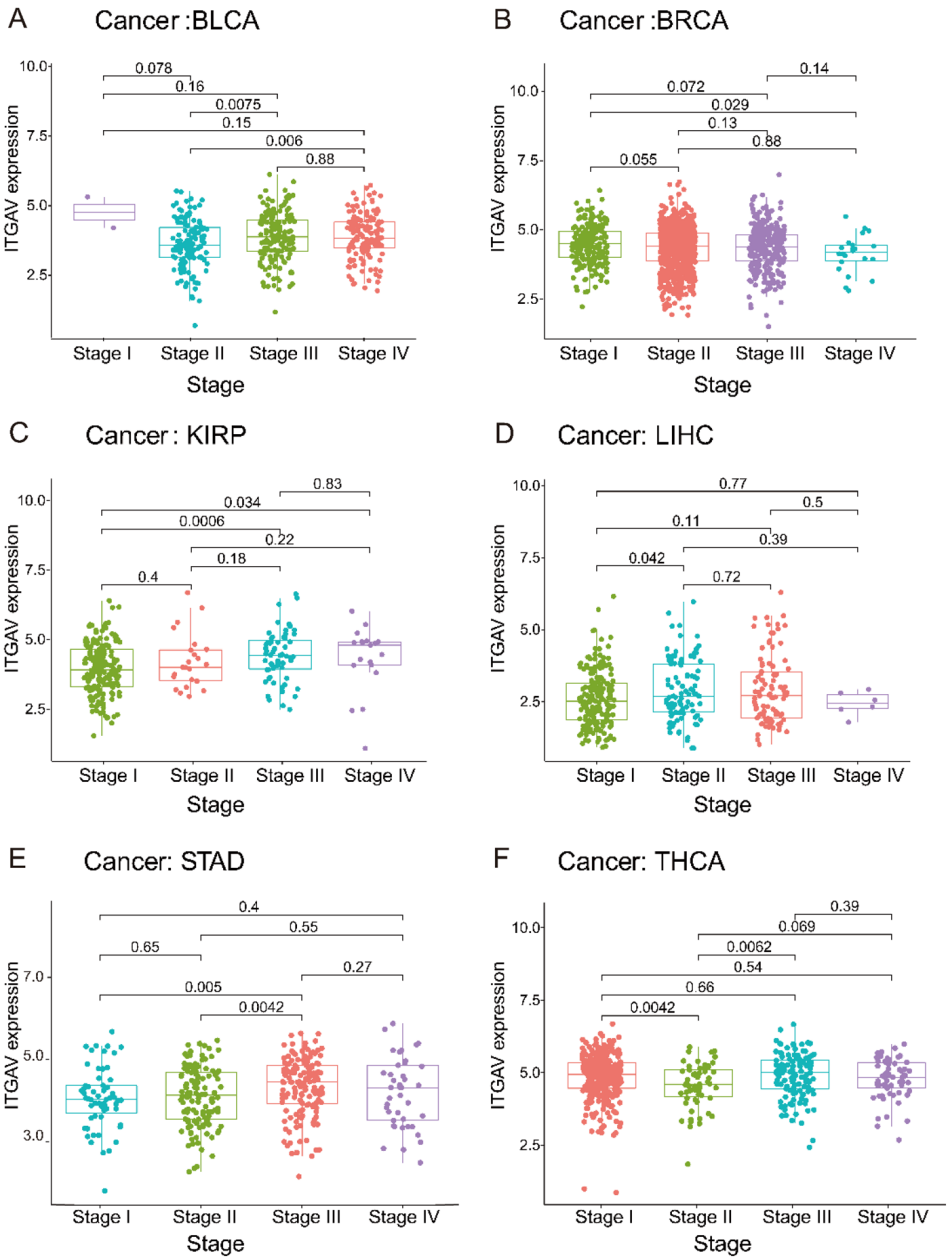
Association between ITGAV expression and clinical stage. (**A**–**F**) Correlation of ITGAV expression with tumor stage in BLCA, BRCA, KIRP, LIHC, STAD, and THCA.

To explore the relationships between ITGAV expression and clinicopathological characteristics, we analyzed data from various cancer types. ITGAV expression was significantly higher in stage IV and III bladder cancer (BLCA) patients than in stage II bladder cancer patients (Fig. 3A). ITGAV expression was higher in stage I breast cancer (BRCA) patients than in stage IV patients (Fig. 3B). In kidney renal papillary cell carcinoma (KIRP), ITGAV expression was lower in stage I patients than in stage III and IV patients (Fig. 3C). ITGAV expression was significantly higher in stage II LIHC than in stage I LIHC (Fig. 3D). In STAD, ITGAV expression was significantly higher in stage III patients than in stage I and II patients (Fig. 3E). Finally, in THCA, ITGAV expression was lower in stage II patients than in stage I and III patients (Fig. 3F). These findings indicate that ITGAV expression significantly varies across different cancer stages, highlighting its potential role in tumor progression and clinical outcomes.

### ITGAV expression is significantly associated with overall survival (OS) across cancers

**Fig. 4:**
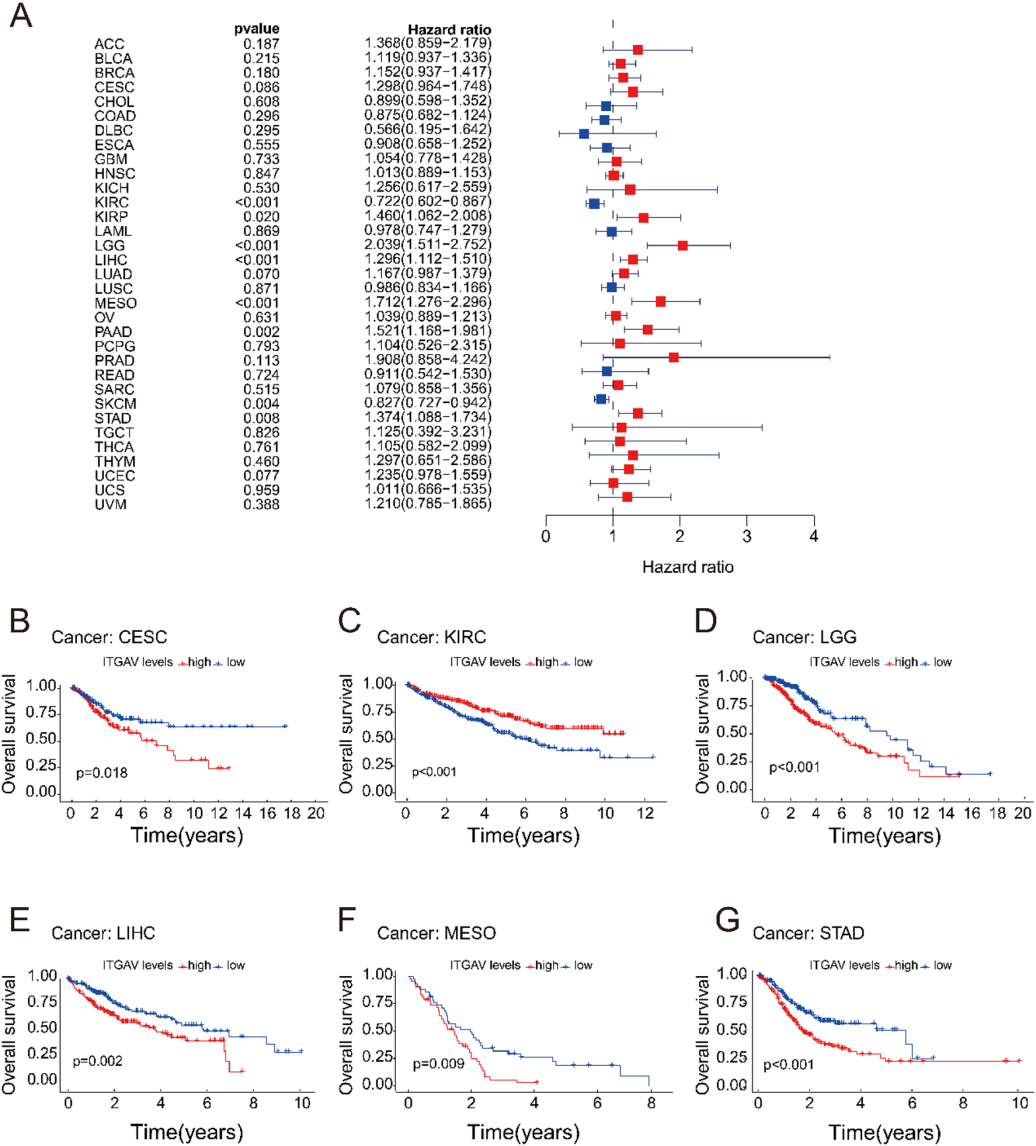
Correlation between ITGAV expression and overall survival (OS). (**A**) Univariate Cox regression analysis of ITGAV expression across cancers, showing HRs. (**B**–**G**) Kaplan–Meier survival curves comparing OS in high vs. low ITGAV expression groups for CESC, KIRC, LGG, LIHC, MESO, and STAD.

We investigated the prognostic value of ITGAV expression in relation to OS across various cancers via data from the TCGA. Univariate Cox regression analysis revealed that ITGAV expression was significantly associated with poor OS in several cancer types, including KIRP, lower-grade glioma (LGG), LIHC, mesothelioma (MESO), and pancreatic adenocarcinoma (PAAD). (Fig. 4A). Among these, LGG had the greatest effect on the risk associated with ITGAV expression (p < 0.05). Further survival analysis revealed that overexpression of ITGAV was associated with shorter OS in most cancer types, except for KIRC, where higher ITGAV expression was correlated with longer survival (Fig. 4B-G). These findings indicate that ITGAV may serve as a potential prognostic biomarker because its expression influences survival outcomes in a tumor type-specific manner.

### ITGAV expression is significantly associated with progression-free survival (PFS) across cancers

**Fig. 5:**
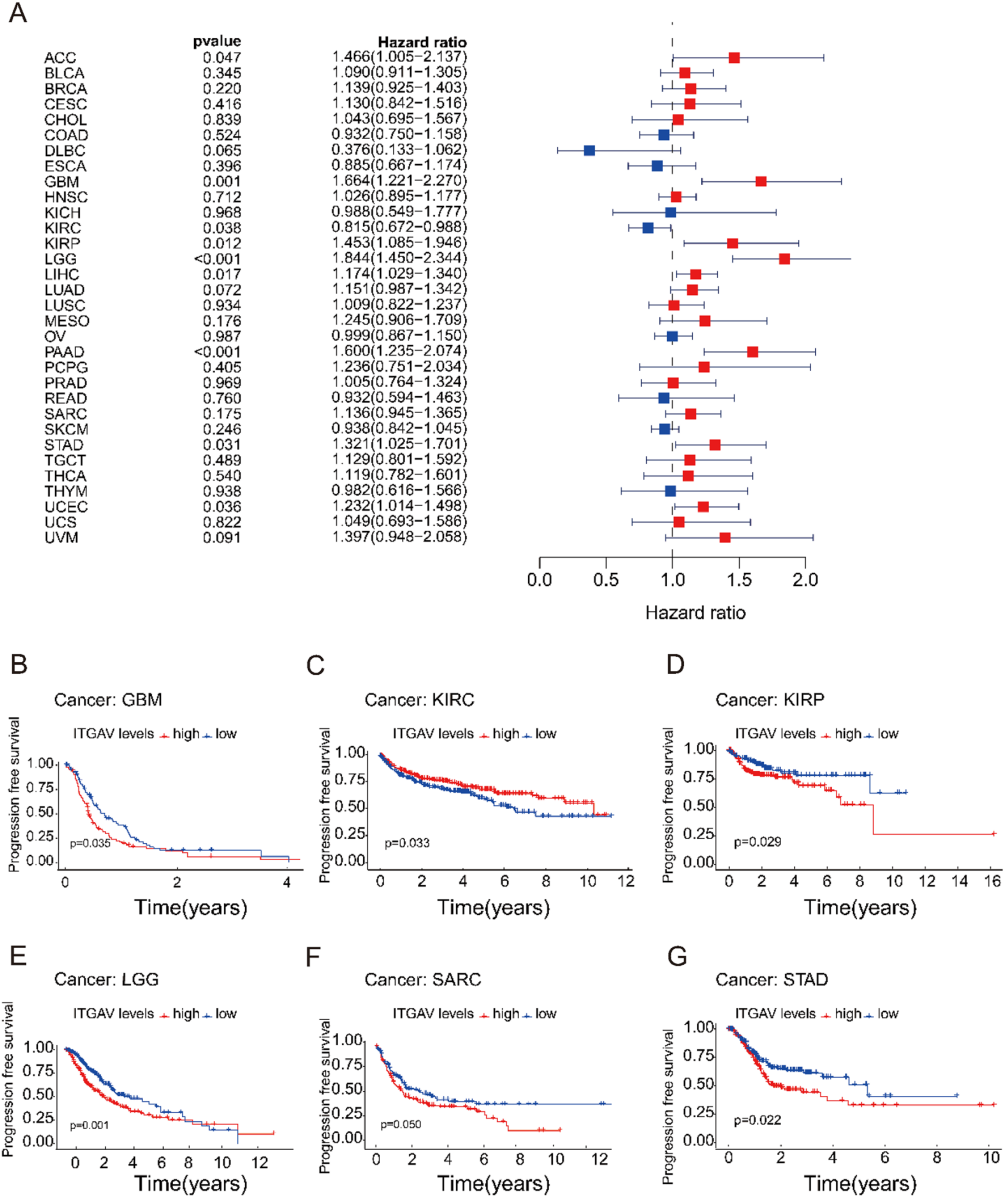
Correlation between ITGAV expression and progression-free survival (PFS). (**A**) Cox regression analysis across cancers demonstrating significant associations between ITGAV expression and PFS. (**B**-**G**) Kaplan–Meier survival curves for GBM, KIRC, KIRP, LGG, SARC, and STAD, comparing PFS between high and low ITGAV expression groups.

We examined the relationship between ITGAV expression and PFS across various cancer types through Cox regression analysis. The analysis revealed significant correlations between ITGAV expression and PFS in several cancers, including adrenocortical carcinoma (ACC), GBM, KIRC, KIRP, LGG, LIHC, PAAD, STAD, and UCEC (Fig. 5A). In general, high ITGAV expression was associated with a shorter PFS, indicating a worse prognosis for patients with high ITGAV expression. However, an exception was observed in KIRC, where higher ITGAV expression was correlated with better PFS (Fig. 5B-G).

### ITGAV expression is significantly associated with disease-specific survival (DSS) in pan-cancer

**Fig. 6:**
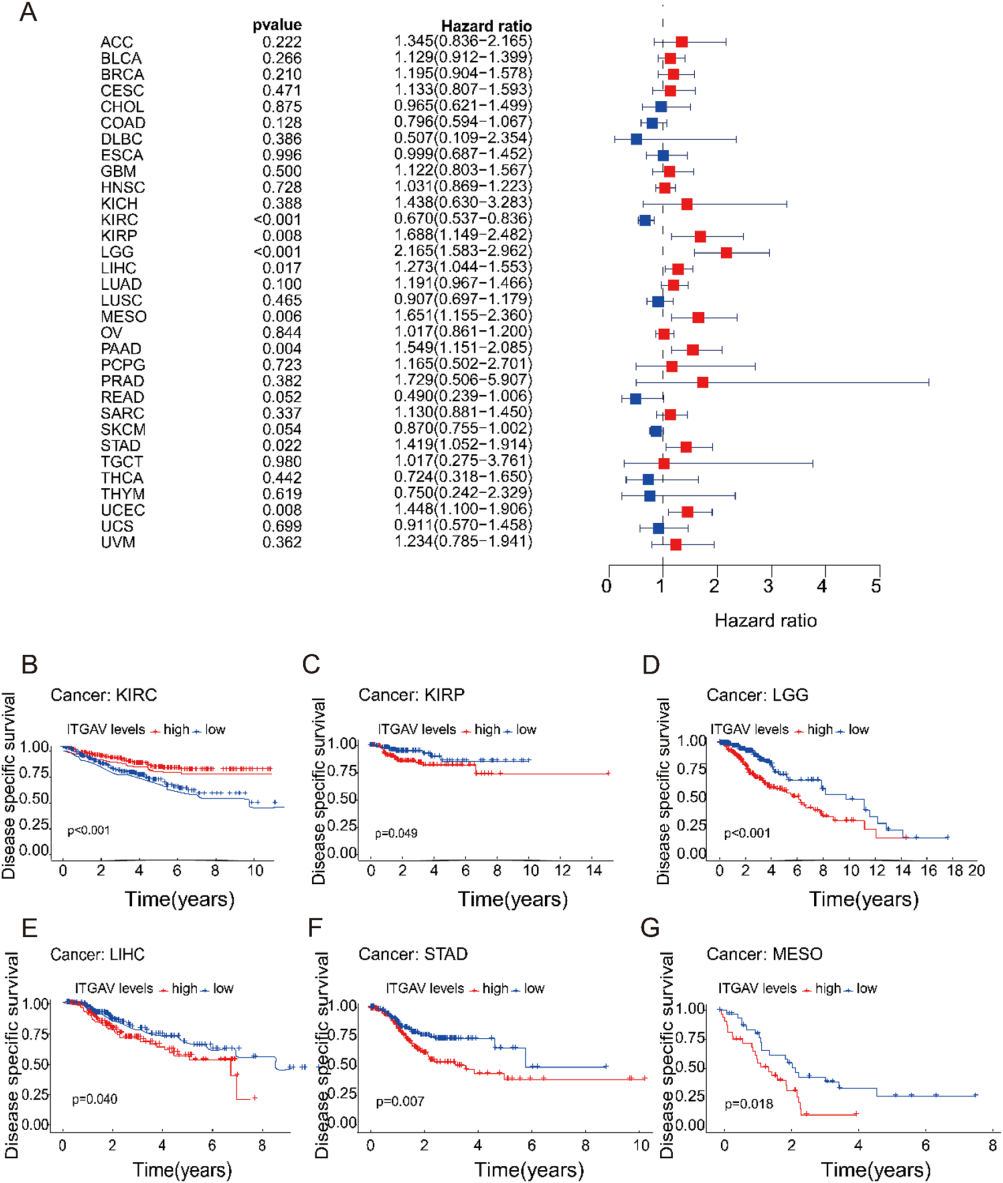
Correlation between ITGAV expression and disease-specific survival (DSS). (**A**) Cox regression analysis revealing associations between ITGAV expression and DSS in multiple cancers. (**B–G**) Kaplan–Meier curves comparing DSS between high and low ITGAV expression groups in KIRC, KIRP, LGG, LIHC, STAD, and MESO.

We conducted Cox regression analysis to assess the relationship between ITGAV expression and DSS across various cancer types. Significant associations (p < 0.05) were observed in KIRC, KIRP, LGG, LIHC, MESO, PAAD, STAD, and UCEC (Fig. 6A). Subsequent survival analysis revealed that, except for KIRC, cancers with high ITGAV expression were associated with lower DSS than those with low expression, suggesting that higher ITGAV levels may be indicative of worse disease-specific survival in most cancer types (Fig. 6B-G).

### ITGAV expression is significantly associated with disease-free survival (DFS) across cancers

**Fig. 7:**
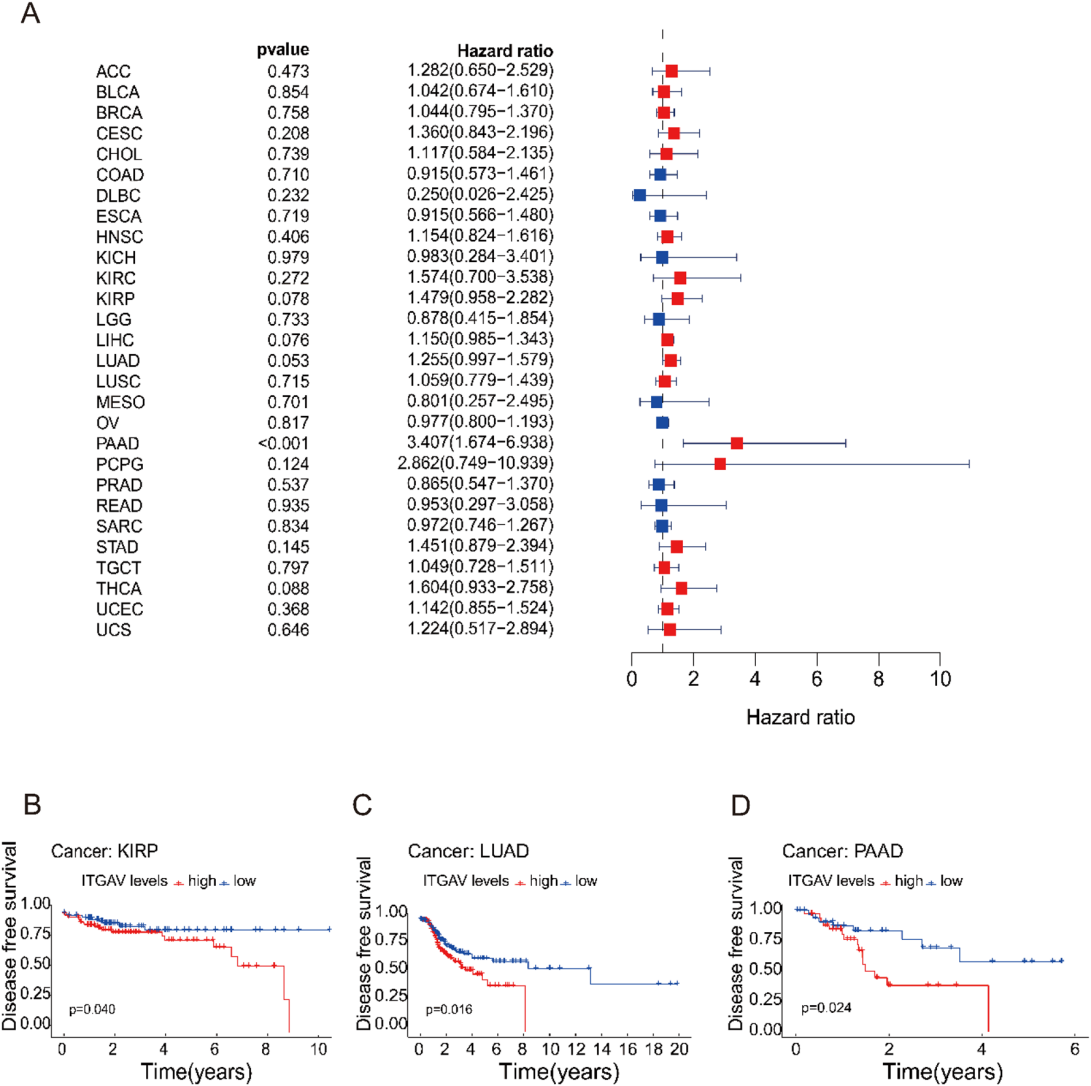
Correlation between ITGAV expression and disease-free survival (DFS). (**A**): Cox regression analysis showing significant associations in PAAD. (**B–D**) Kaplan–Meier analysis showing differences in DFS between high and low ITGAV expression in KIRP, LUAD, and PAAD.

To explore the relationship between ITGAV expression and DFS across various cancer types, we performed Cox regression analysis. Notably, ITGAV expression was found to significantly affect DFS in patients with PAAD (p < 0.05) (Fig. 7A). Survival analysis revealed that patients with high ITGAV expression had a worse DFS than those with low ITGAV expression did, indicating that ITGAV may serve as a negative prognostic factor in PAAD (Fig. 7B-D).

### PPI Network and enrichment analysis for pan-cancer

**Fig. 8:**
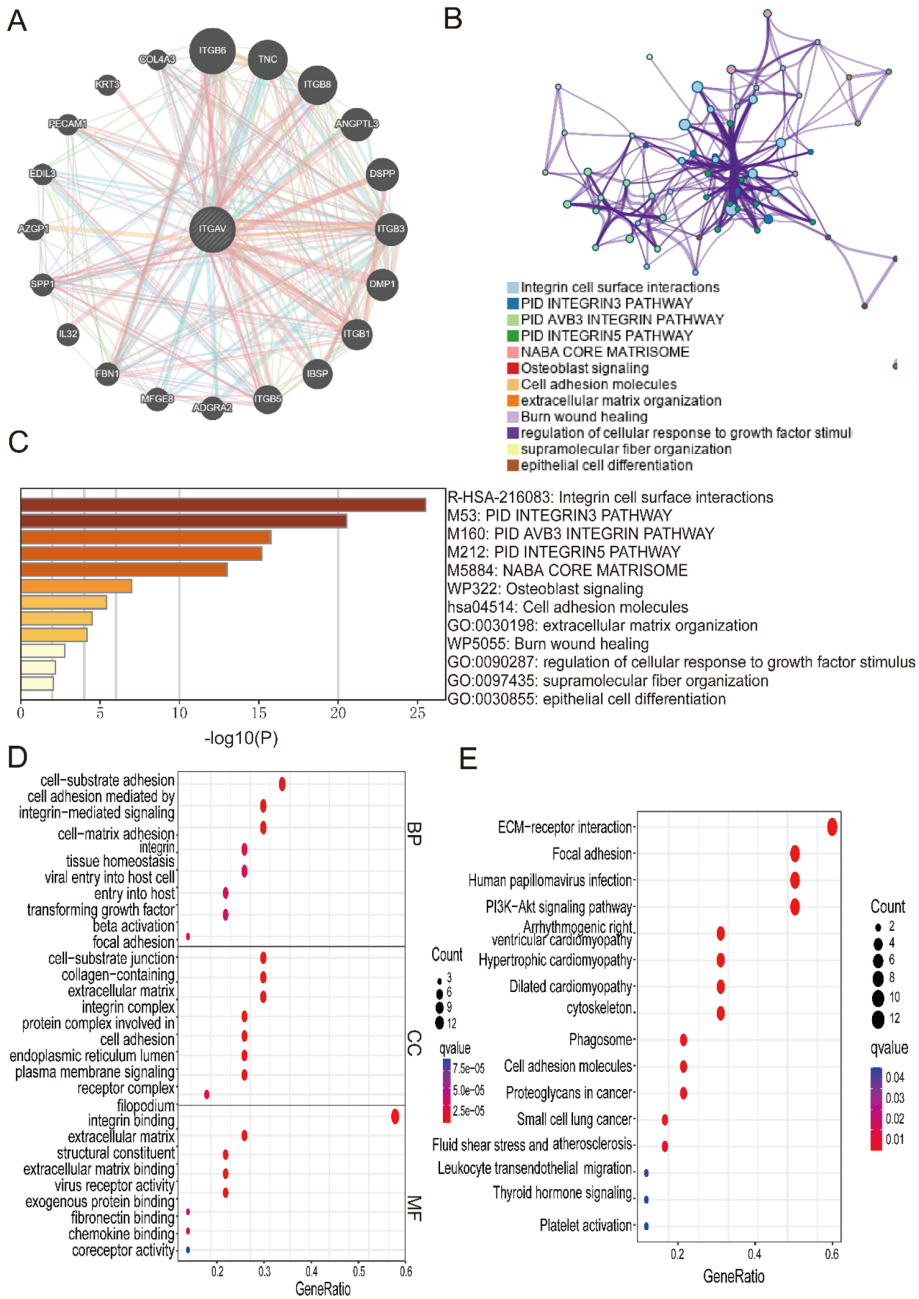
PPI network and enrichment analysis of ITGAV. (**A**) GeneMANIA-derived PPI network illustrating interactions between ITGAV and proteins such as ITGB6, TNC, and SPP1.(**B–E**) GO and KEGG enrichment analyses revealing ITGAV involvement in integrin signaling, ECM–receptor interaction, and PI3K–Akt pathways.

PPI network analysis via GeneMANIA revealed that ITGAV interacts with several key proteins, including ITGB6, TNC, ITGB8, ANGPTL3, DSPP, ITGB3, DMP1, ITGB1, IBSP, ITGB5, and SPP1 (Fig. 8A). These findings suggest that ITGAV is involved in multiple molecular and cellular processes. The functional enrichment analysis revealed several enriched pathways and biological functions associated with ITGAV and its interacting partners. These pathways primarily included integrin cell surface interactions, the integrin3 pathway, osteoblast signaling, extracellular matrix organization, and epithelial cell differentiation (Figs. 8B-C). Gene Ontology analysis revealed enriched molecular functions related to integrin binding, extracellular matrix binding, chemokine binding, and coreceptor activity. The enriched cellular components were linked to focal adhesion, cell-substrate junctions, and extracellular matrix-related structures (Fig. 8D). The enriched biological processes were associated with cell–matrix adhesion, integrin-mediated signaling, tissue homeostasis, and viral entry into host cells. Further analysis via KEGG pathway analysis revealed significant enrichment of pathways related to proteoglycans in cancer, ECM-receptor interaction, focal adhesion, and the PI3K-Akt signaling pathway, as well as pathways linked to human papillomavirus infection and heart-related conditions such as arrhythmogenic right ventricular cardiomyopathy (Fig. 8E). These results suggest that ITGAV may play crucial roles in cancer progression through interactions that affect cell adhesion, signaling, and extracellular matrix remodeling.

### ITGAV expression is significantly associated with a wide range of immune cells in pan-cancer

**Fig. 9:**
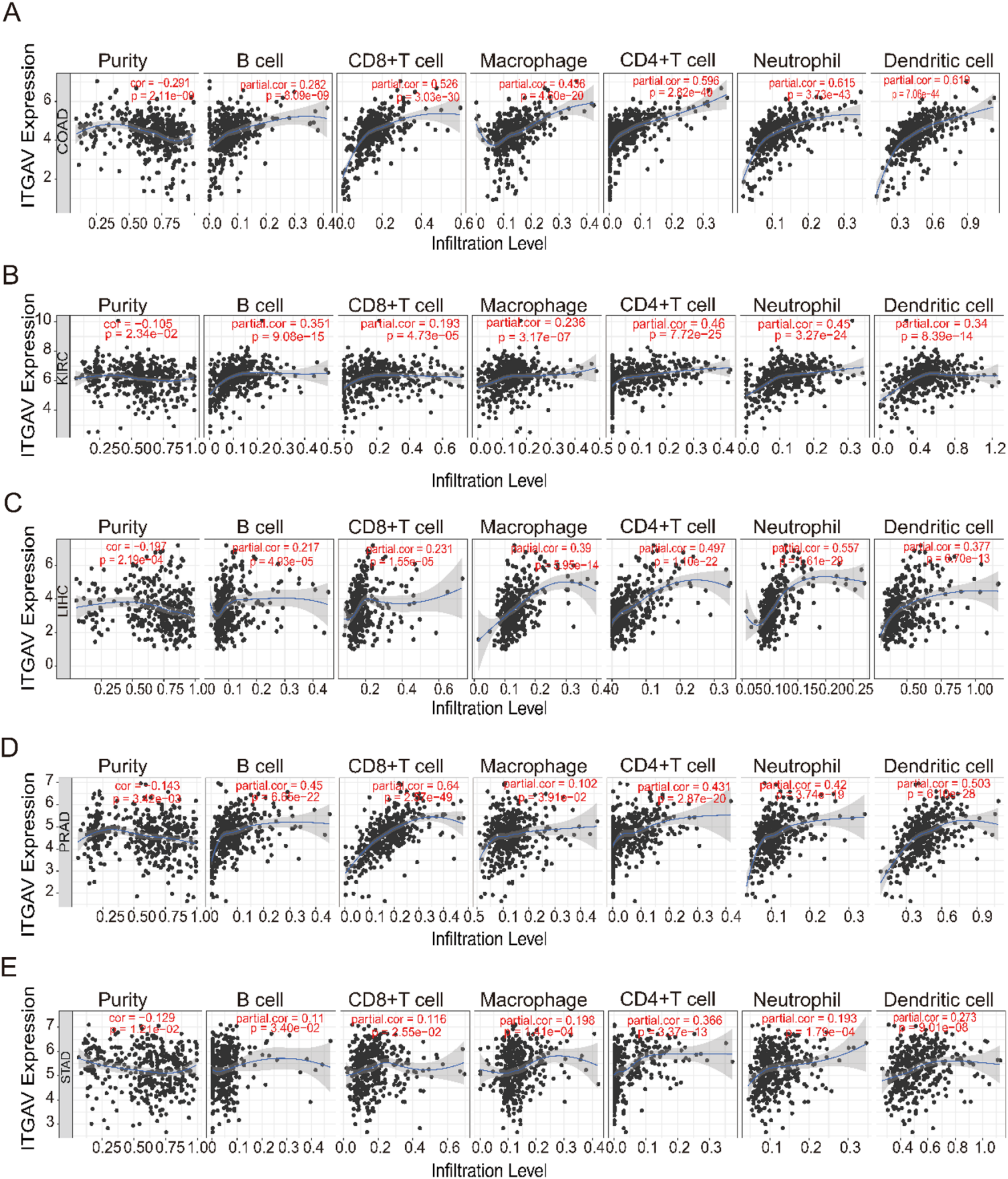
Correlation between ITGAV expression and immune infiltration. (**A–E**) Correlation analysis between ITGAV expression and infiltration of immune cells in COAD, KIRC, LIHC, PRAD, and STAD. Both positive and negative correlations are shown, highlighting the potential immunological role of ITGAV.

Our analysis revealed significant correlations between ITGAV expression and immune cell infiltration across various cancer types. Figures 9A-E show that in COAD, KIRC, LIHC, prostate adenocarcinoma (PRAD), and STAD, ITGAV expression levels were negatively correlated with tumor purity. These findings suggest that ITGAV expression is closely linked to immune cell infiltration in most cancers, particularly in COAD, KIRC, LIHC, PRAD, and STAD, where higher ITGAV expression is correlated with greater immune cell infiltration. Overall, these results highlight the potential of ITGAV as an important factor influencing the immune microenvironment and its role in cancer progression.

### Significant correlation between ITGAV expression and immune-related genes in pan-cancer

**Fig. 10:**
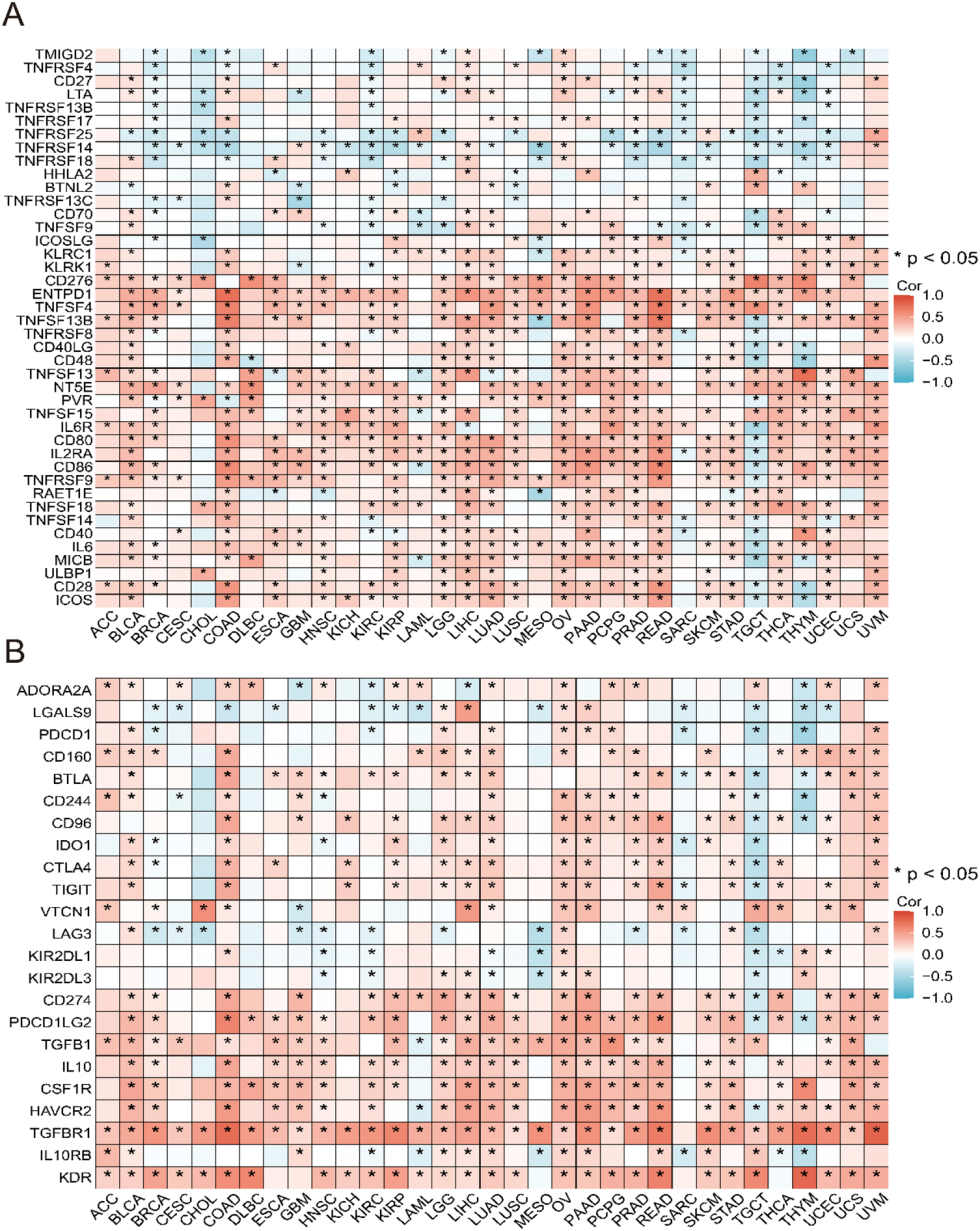
Correlation between ITGAV and immune regulatory genes. (**A**) Heatmap showing correlation between ITGAV and immune stimulatory genes across cancers. (**B**) Heatmap showing correlation between ITGAV and immune suppressive genes. Statistically significant results are marked with asterisks.

Furthermore, we performed co-expression analysis across 33 cancer types to explore the relationships between ITGAV expression and immune-related genes. Figures 10A-B show the correlations between ITGAV and immune activation genes and immunosuppressive genes. Supplementary Material Figure S1 provides further insights into the relationships between ITGAV and immune checkpoint genes as well as MHC genes, while Supplementary Material Figure S2 highlights the associations between ITGAV and chemokines along with their receptors. The heatmap shows that most immune-related genes are significantly co-expressed with ITGAV across various cancer types. These findings suggest that ITGAV may play a crucial role in regulating immune cell infiltration, thereby influencing the therapeutic efficacy of ICIs and serving as a key immune modulator.

### Molecular docking analysis of ITGAV with six potential therapeutic drugs

**Fig. 11:**
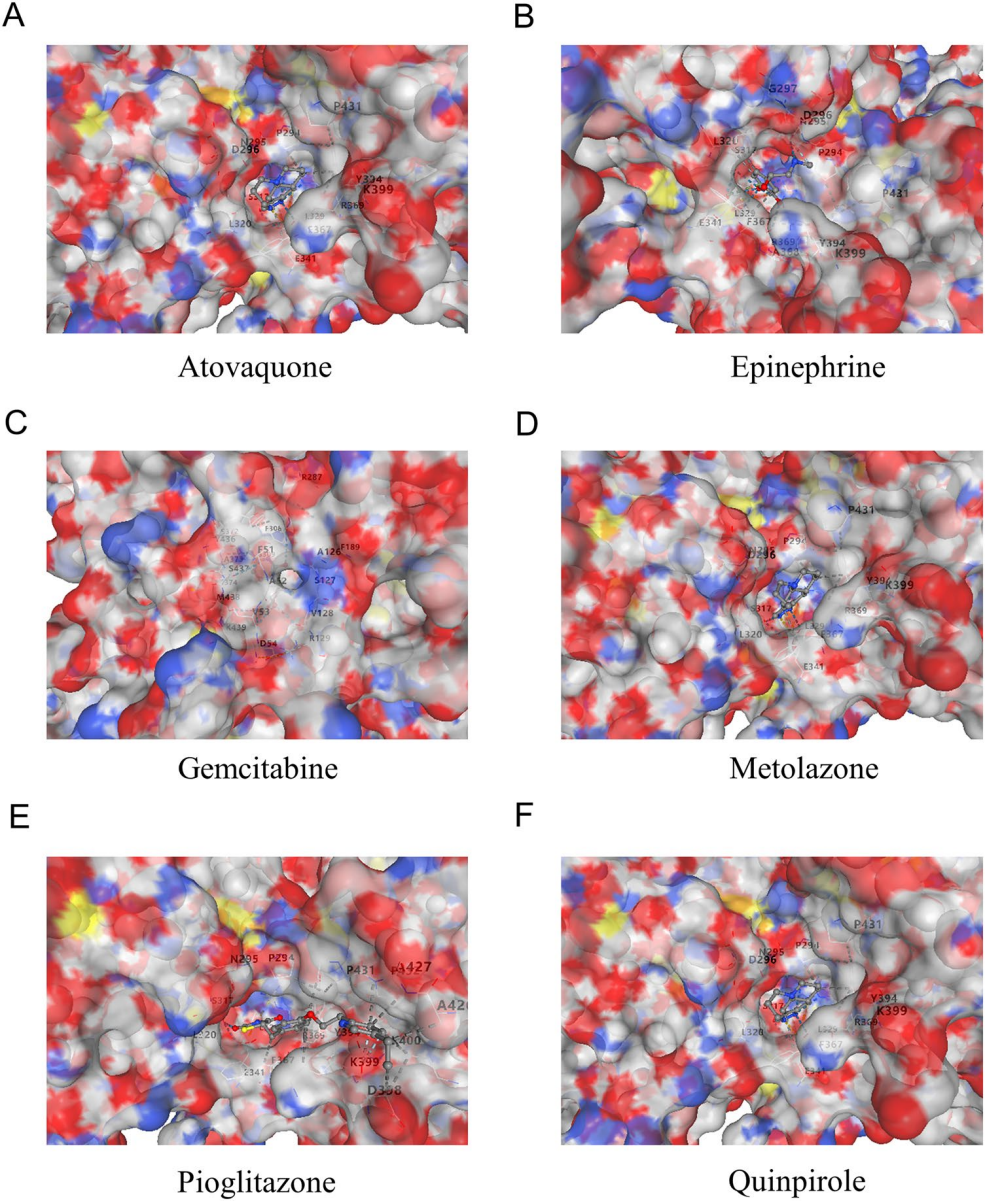
Molecular docking of ITGAV with selected drugs. (**A**–**F**). Docking of six candidate drugs (Atovaquone, Epinephrine, Gemcitabine, Metolazone, Pioglitazone, Quinpirole) with ITGAV. Binding sites and key interacting residues are visualized for each compound.

The molecular docking results revealed interactions between ITGAV and six different drugs: atovaquone (Fig. 11A), epinephrine (Fig. 11B), gemcitabine (Fig. 11C), metolazone (Fig. 11D), pioglitazone (Fig. 11E), and quinpirole (Fig. 11F). Each panel displays the three-dimensional surface of ITGAV with the corresponding drug molecule accurately docked into its binding pocket. An analysis of the positions of key residues and binding affinities revealed how these drugs bind to ITGAV and potentially modulate its function. These findings suggest that ITGAV could serve as a promising therapeutic target, with the identified drugs potentially offering new avenues for modulating ITGAV-related pathways in cancer treatment.

### ITGAV expression is associated with immunotherapy response potential in multiple cancer types

**Fig. 12:**
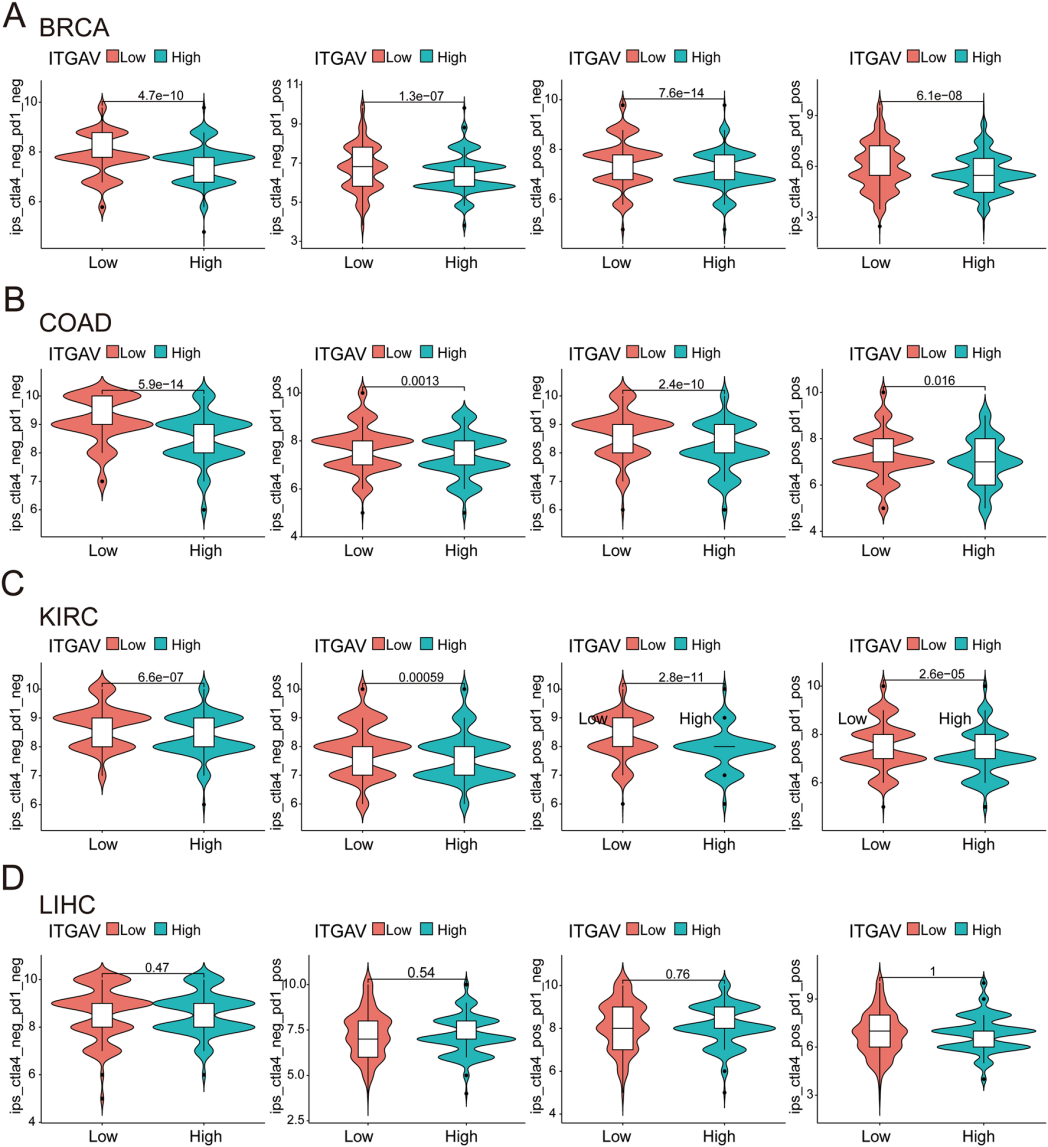
Correlation of ITGAV expression with immunotherapy efficacy across cancer types. (**A**) In BRCA, low ITGAV expression is associated with significantly higher IPS across all four immune checkpoint inhibitor (ICI) conditions, indicating greater predicted sensitivity to immunotherapy. (**B**) In COAD, low ITGAV expression also correlates with improved immunotherapy response across all IPS categories.(**C**) In KIRC, lower ITGAV expression is significantly associated with higher IPS in most ICI settings. (**D**) In LIHC, no significant difference in IPS is observed between high and low ITGAV expression groups under any ICI condition.

To further assess the predictive value of ITGAV in immunotherapy, we analyzed immunophenoscores (IPS) from the TCIA database. In BRCA, COAD, and KIRC, tumors with low ITGAV expression exhibited significantly higher IPS across all four immune checkpoint blockade scenarios (Fig. 12A–C), indicating a potentially stronger responsiveness to immunotherapy. In contrast, no significant differences were observed in LIHC between high and low ITGAV expression groups (Fig. 12D). These findings suggest that ITGAV may serve as a context-dependent biomarker for predicting immunotherapy efficacy in selected cancer types.

## Discussion

In this study, we conducted the first comprehensive pan-cancer analysis of ITGAV expression and its significance via data from the TCGA, GTEX, CCLE, and THPA databases. Our findings offer a systematic and in-depth exploration of ITGAV expression at multiple tissue and cellular levels, shedding light on its correlation with tumor diagnosis, prognosis, clinicopathological characteristics, cancer-related pathways, immune cell infiltration, immune-related genes, and immunotherapy responses. This analysis has significantly advanced our understanding of the ITGAV signature across various cancers, suggesting promising prospects for the development of targeted inhibitors and immunotherapeutic strategies against ITGAV. Furthermore, these findings provide a robust foundation for future research into the role of ITGAV in cancer pathogenesis, paving the way for more effective therapeutic interventions.

At the tissue level, analyses from the TIMER and TCGA databases revealed that ITGAV was significantly upregulated in a range of cancers, including CHOL, ESCA, HNSC, LIHC, LUAD, LUSC, STAD and THCA. Notably, ITGAV overexpression has been prominently observed in LIHC and BRCA^41,42^. Additionally, ITGAV was found to be upregulated in PAAD, COAD, and STAD, where its elevated expression is closely associated with poor prognosis^43–45^. Our findings further underscore that ITGAV expression is notably increased in COAD, LIHC, and PAAD, whereas it is significantly downregulated in kidney cancer tissues. These findings suggest that ITGAV may play a critical role in the progression of certain cancers, whereas its lower expression in kidney cancer highlights potential differences in its functional significance across tumor types.

Through Cox regression and Kaplan‒Meier analyses, we found that ITGAV expression is closely linked to tumor progression and poor prognosis in various malignant solid tumors. For example, elevated ITGAV expression was associated with decreased OS in patients with CESC, LGG, LIHC, MESO, and STAD. Furthermore, it influenced PFS in GBM, KIRP, LGG, SARC, and STAD patients. ITGAV expression was also correlated with poorer DSS in LGG, LIHC, STAD, and MESO patients. Notably, the expression of ITGAV was significantly associated with only DFS in PAAD patients. Interestingly, in KIRC patients, higher ITGAV expression was linked to better OS, PFS, and DSS, although the underlying mechanisms remain unclear and warrant further exploration in future studies. These findings underscore the critical role of ITGAV as a potential prognostic biomarker, highlighting its diverse impact on survival outcomes across various cancer types and emphasizing the need for further research to fully elucidate its mechanisms and therapeutic implications.

This study establishes a robust connection between ITGAV expression and immune cell infiltration across cancers such as COAD, LIHC, PRAD, and STAD, underscoring its pivotal role in modulating the TME. This finding aligns with a growing body of evidence highlighting the critical role of integrin-mediated cell adhesion and signaling in immune cell recruitment and activation, ultimately shaping the immune landscape of tumors^46,47^. The negative correlation between ITGAV expression and tumor purity further suggests that elevated ITGAV levels may foster a more immune-enriched microenvironment, potentially improving patient prognosis and increasing the efficacy of immunotherapy, especially in COAD and PRAD. This finding is in agreement with prior research emphasizing the central role of immune infiltration in determining the success of immunotherapy^48,49^.

Moreover, ITGAV expression is intricately linked to a variety of immune-related genes, including those governing immune activation and immune checkpoint regulation. The positive correlation between ITGAV and immune checkpoint genes indicates its potential involvement in immune evasion, thereby influencing the effectiveness of ICIs. These findings are consistent with those of previous studies that highlighted the importance of integrins and ECM components in immune regulation, suggesting that these molecules are promising targets for enhancing immunotherapy outcomes^50,51^. These findings position ITGAV as a promising biomarker for predicting immunotherapy efficacy and offer valuable insights into advancing immune-based cancer treatments. Further research into the molecular mechanisms of ITGAV could pave the way for the development of more effective, targeted, and personalized immunotherapies.

To further explore the potential of ITGAV in pan-cancer therapy, we evaluated its ability to bind several drugs via databases such as DSigDB, PubChem, and PDB. The results revealed that ITGAV strongly binds several small-molecule drugs, including atovaquone, epinephrine, gemcitabine, metolazone, pioglitazone, and quinpirole, suggesting that ITGAV may serve as a promising target to modulate the therapeutic efficacy of these drugs. Of particular interest is gemcitabine, a well-established chemotherapeutic agent widely used to treat pancreatic cancer, lung cancer, breast cancer, and other malignant tumors^52–55^. Its interaction with ITGAV further underscores the potential of ITGAV in cancer therapy, highlighting that by targeting the molecules it interacts with, drug efficacy could be enhanced or new therapeutic pathways may be explored. These findings provide a solid foundation for the future development of personalized targeted therapies based on ITGAV and offer new possibilities for precision medicine in cancer treatment.

Immunotherapy has emerged as a highly promising treatment strategy, harnessing the body’s immune system to combat cancer cells^56,57^. Among ICIs, those that target PD-1 and CTLA-4 have been widely adopted in clinical practice, offering significant therapeutic benefits for many cancer patients^58,59^. Our analysis of IPS from the TCIA database revealed that patients with low ITGAV expression in several common malignancies, including BRCA, COAD, and KIRC, demonstrated significantly higher IPS under multiple ICI conditions, suggesting a stronger potential response to immune checkpoint blockade. However, this trend was not observed in LIHC, where ITGAV expression did not significantly stratify IPS scores, indicating its context-dependent predictive value. These findings highlight the utility of ITGAV as a predictive biomarker for immunotherapy response, rather than a universal enhancer of immune efficacy. Further mechanistic studies are warranted to explore the biological basis underlying these associations and to validate ITGAV’s role in clinical immunotherapy stratification.

In conclusion, our findings suggest that ITGAV can be utilized as an independent prognostic factor for a number of cancers and that its expression level predicts similar results across malignant tumors. In addition, additional research into the special role of ITGAV in each type of cancer is needed. These results may help clarify the role of ITGAV in intercellular delivery and tumor promotion and provide a foundation for more targeted and personalized ICIs in the future.

## Supplementary Information

Supplementary Information.

## Data Availability

The data and R scripts used in this study are available upon request from the corresponding author.

## Abbreviations

ACC: Adrenocortical carcinoma
AUC: Area Under the Curve
BLCA: Bladder cancer
BRCA: Breast cancer
CCLE: The Broad Institute Cancer Cell Line Encyclopedia
CESC: Cervical squamous cell carcinoma and endocervical adenocarcinoma
CHOL: Cholangiocarcinoma
COAD: Colon adenocarcinoma
DEGs: Differentially expressed genes
DFI: Disease-free interval
DFS: Disease-free survival
DSS: Disease-specific survival
ECM: Extracellular matrix
ESCA: Esophageal carcinoma
ESCC: Esophageal squamous cell carcinoma
GBM: Glioblastoma multiforme
GEO: Gene Expression Omnibus
GO: Gene Ontology
GTEx: Genotype-Tissue Expression
HNSC: Head and neck squamous cell carcinoma (HNSC)
ICI: Immune checkpoint inhibitor
IPS: Immunophenotype Scores
KEGG: Kyoto Encyclopedia of Genes and Genomes
KICH: Kidney chromophobe
KIRC: Kidney renal clear cell carcinoma
KIRP: Kidney renal papillary cell carcinoma
LGG: Lower-grade glioma
LIHC: Liver hepatocellular carcinoma
LUAD: Lung adenocarcinoma
LUSC: Lung squamous cell carcinoma
MESO: Mesothelioma
MHC: Major histocompatibility complex
OS: Overall survival
PAAD: Pancreatic adenocarcinoma
PBS: Phosphate buffered saline
PCA: Principal component analysis
PFI: Progression-free interval
PFS: Progression-free survival
PPI: Protein‒protein interactions
PRAD: Prostate adenocarcinoma
ROC: Receiver operating characteristic
SCC: Squamous cell carcinoma
SKCM: Skin cutaneous melanoma
STAD: Stomach adenocarcinoma
TCGA: The Cancer Genome Atlas
TCIA: The Cancer Immunome Database
THCA: Thyroid carcinoma
THPA: Human Protein Atlas
TIMER: The Tumor Immune Estimation Resource
TME: The tumor microenvironment
UCEC: Uterine corpus endometrial carcinoma
UMIs: Unique molecular identifiers
