# Supplementary material for: ITGAV as a promising diagnostic, immunological, and prognostic biomarker in pan-cancer
Source: Sci Rep. 2025 Aug 7;15:28942. doi: 10.1038/s41598-025-11836-8 (PMC12332088; doi:10.1038/s41598-025-11836-8)
Supplement: Supplementary file 1 — Supplementary Information. [file 41598_2025_11836_MOESM1_ESM.docx]

**Supplementary material**

**Figure S1**


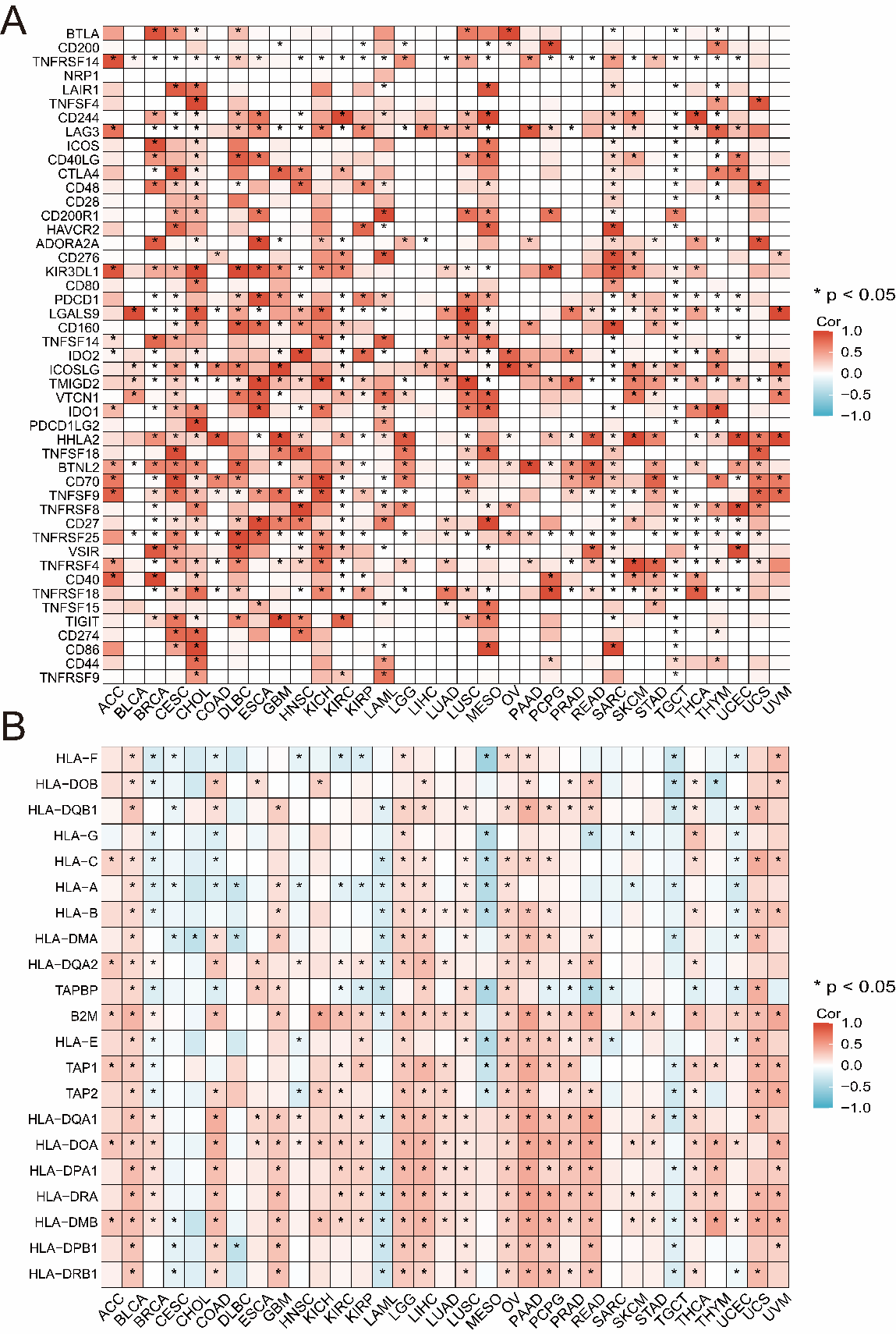


**Figure S1: Correlations of ITGAV expression with immune checkpoint genes and major histocompatibility complex (MHC) genes in pan-cancer**

**A:** Heatmap illustrating the correlation between ITGAV expression and immune checkpoint genes across various cancer types. Positive correlations are represented in red, and negative correlations are represented in blue. Significant correlations (*p < 0.05) are marked with asterisks.

**B:** Heatmap showing the correlation between ITGAV expression and major histocompatibility complex (MHC) genes across cancers. As in Panel A, positive correlations are shown in red, and negative correlations are shown in blue, with significant correlations (*p < 0.05) indicated by asterisks. These results highlight the widespread association of ITGAV with immune checkpoint and HLA genes, which play critical roles in the immune response to cancer.

**Figure S2**


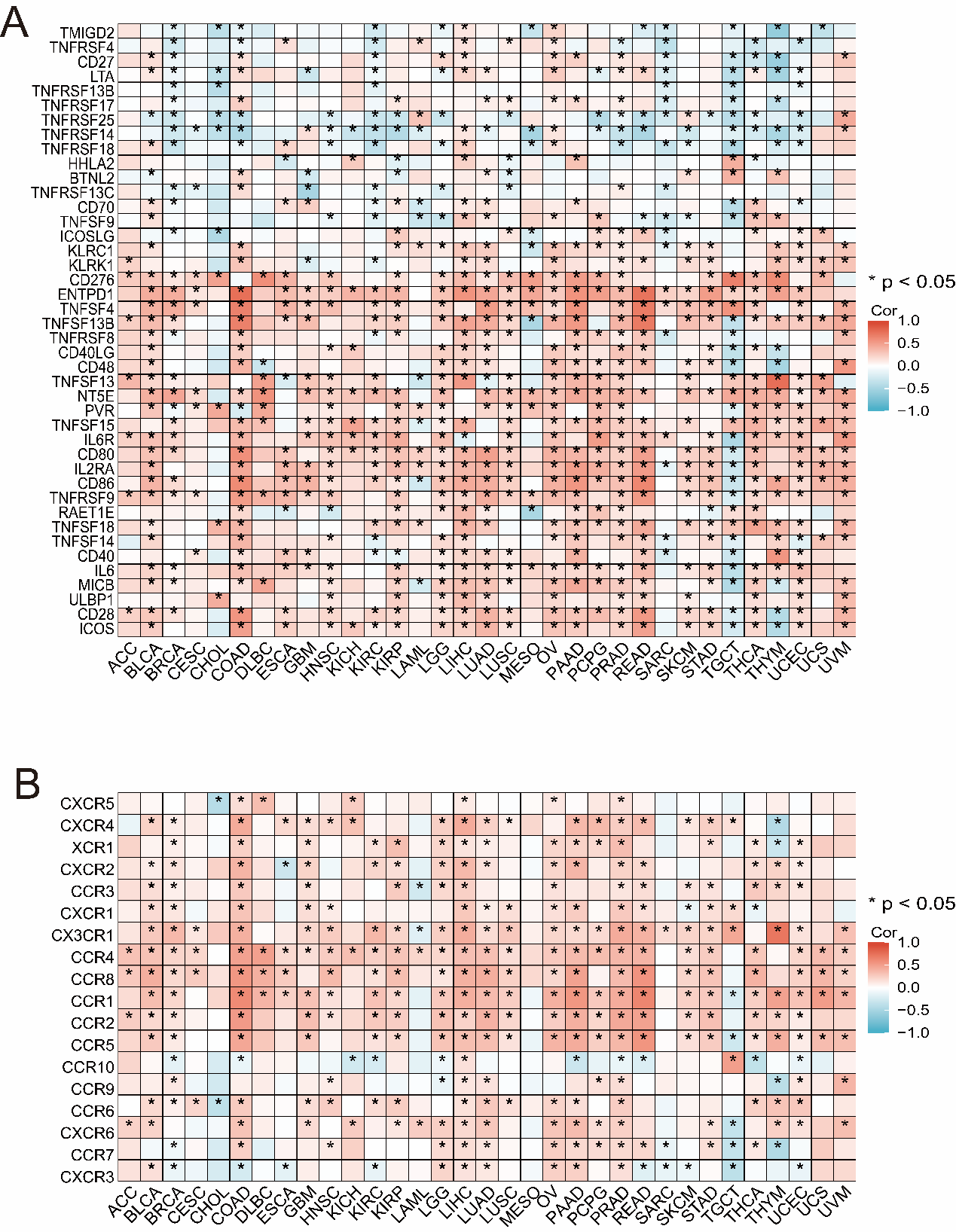


**Figure S2: Correlation of ITGAV expression with cytokines and cytokine receptors across cancers**
**A**: Heatmap showing the correlation between ITGAV expression and cytokines across various cancer types. Positive correlations are represented in red, and negative correlations are represented in blue. Significant correlations (*p < 0.05) are marked with asterisks.
**B**: Heatmap illustrating the correlation between ITGAV expression and cytokine receptor expression across cancers. In Panel A, red indicates positive correlations, blue indicates negative correlations, and significant correlations (*p < 0.05) are indicated by asterisks. These results highlight the relationship between ITGAV and immune signaling molecules, which may have important implications for immune response regulation in cancer.
